# Tetrahydrobiopterin and Autism Spectrum Disorder: A Systematic Review of a Promising Therapeutic Pathway

**DOI:** 10.3390/brainsci15020151

**Published:** 2025-02-03

**Authors:** Clóvis Colpani Filho, Lucas Melfior, Sthephanie Luiz Ramos, Mateus Santos Oliveira Pizi, Lilian Freitas Taruhn, Margrit Ellis Muller, Thiago Kucera Nunes, Luísa de Oliveira Schmitt, Joana Margarida Gaspar, Miguel de Abreu de Oliveira, Giovanna Tassinari, Luisa Cruz, Alexandra Latini

**Affiliations:** 1 Laboratório de Bioenergética e Estresse Oxidativo—LABOX, Departamento de Bioquímica, Universidade Federal de Santa Catarina, Florianópolis 88040-900, Brazil; 2 Medicine School, Universidade Federal de Santa Catarina, Florianópolis 88040-900, Brazil; 3 Pharmacy School, Universidade Federal de Santa Catarina, Florianópolis 88040-900, Brazil

**Keywords:** autism, neopterin, nitric oxide, neurotransmitters, inflammation

## Abstract

Autism Spectrum Disorder (ASD) is a neurodevelopmental condition characterized by persistent deficits in social communication and interaction, along with restricted and repetitive patterns of behavior, interests, or activities. ASD encompasses a wide spectrum of clinical presentations and functional impairments, ranging from mild to severe. Despite its prevalence, the underlying physiopathological mechanisms of ASD remain largely unknown, resulting in a lack of effective targeted therapeutic interventions, contributing to significant financial and emotional burdens on affected families and the healthcare system. Emerging evidence suggests that dysfunction in the tetrahydrobiopterin (BH4) pathway may impair the activity of monoaminergic and nitric oxide (NO)-dependent neurons in individuals with ASD. To explore this potential mechanism, we conducted a systematic review to analyze such impairments to gather information on whether the off-label use of BH4 could represent a novel pharmacological approach for managing ASD. Following the PRISMA 2020 guidelines, we systematically reviewed the literature from four databases: PubMed, Virtual Health Library, Cochrane Library, and SciELO, from January 1967 to December 2021. The quality of the included studies was assessed using the Newcastle–Ottawa scale. The inclusion criteria for this systematic review focused on identifying articles published in English that contained the following keywords, used in various combinations: *autism*, *ASD*, *autism spectrum disorder*, *BH4, tetrahydrobiopterin*, *neopterin*, *NO*, *nitric oxide*. The analysis was performed between December 2020 and December 2021. The collected data demonstrated that BH4 metabolism was altered in individuals with ASD. Lower levels of BH4 were reported in biological samples from ASD-affected individuals compared to age- and sex-matched controls. Additionally, neopterin levels were elevated in plasma and urine, but decreased in cerebrospinal fluid, while nitric oxide levels were consistently reported to be higher across studies. Treatment with BH4 has shown potential in improving ASD-related symptoms. The reported increase in neopterin in biological fluids indicates inflammation, while the reduction in BH4 levels suggests a potential shift in its metabolic role. Specifically, BH4 may be diverted from its primary role in neurotransmitter synthesis to function as an antioxidant or to perpetuate inflammation through NO production. Given that BH4 is a critical cofactor in monoaminergic neurotransmission, its dysfunction highlights the molecule’s therapeutic potential. BH4, already FDA-approved for other conditions, emerges as a promising off-label candidate to alleviate ASD symptomatology.

## 1. Introduction

Autism Spectrum Disorder (ASD) is a neurodevelopmental condition characterized by persistent deficits in three domains: social interaction, communication, and repetitive/restricted behaviors [1]. ASD encompasses a wide spectrum of clinical presentations and functional impairments, ranging from mild to severe. Due to the complexity, severity, and overlap of ASD symptoms with other psychiatric disorders, several clinical tools and behavioral assessments are needed for the correct diagnosis, including parent/caregiver interviews, patient interviews, direct observation of patients, and detailed clinical assessments that encompass a thorough review of family history of ASD or other neurodevelopmental disorders.

The prevalence of ASD is 17 and 18.5 cases per 1000 children in the United States aged 4 and 8 years, respectively, while in Europe, it ranges between 3.8 and 15.5 cases per 1000 individuals [2]; however, this prevalence may be underestimated due to challenges in diagnosis and the absence of reliable, sensitive, and specific quantifiable biomarkers.

The pathogenesis of ASD is not completely understood, but involves a combination of genetic and environmental factors, persistent inflammation, and immune dysregulation. For example, various mutations and polymorphisms in genes regulating synaptic function, neurotransmitter pathways, and immune responses have been strongly implicated [3,4]. Indeed, neurobiological hallmarks of ASD include abnormal synaptic connectivity, imbalances in excitatory–inhibitory signaling, and alterations in neurotransmitter systems such as serotonin, dopamine, glutamate, and gamma aminobutiric acid (GABA) [4,5]. Immune dysfunction, chronic inflammation, oxidative stress, and metabolic abnormalities, including mitochondrial dysfunction, have been described as critical factors [6,7,8,9,10]. Considering that many of these molecular and signaling pathways may be dependent on the availability of 6R-L-erythro-5,6,7,8-tetrahydrobiopterin (BH4), alterations in its metabolism have been implicated in the pathogenesis of ASD.

### 1.1. BH4 Metabolism: Biosynthesis and Regeneration

**Figure 1:**
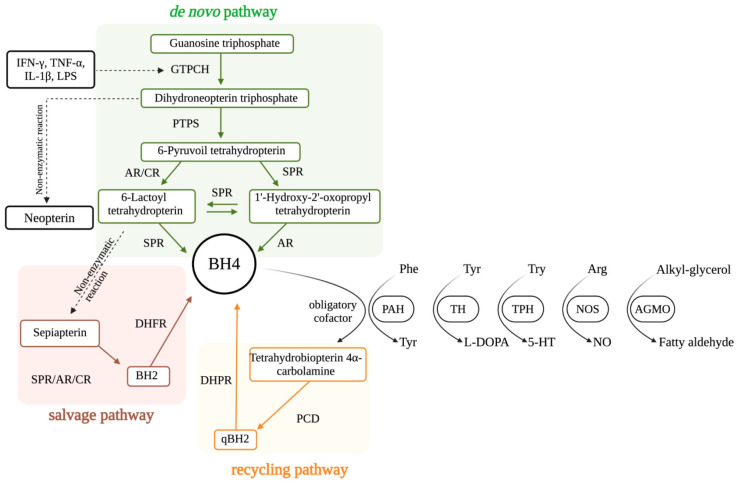
Metabolic routes involved in tetrahydrobiopterin (BH4) biosynthesis. BH4 is a mandatory cofactor for the catalytic activity of five enzymes, namely phenylalanine hydroxylase (PAH), tyrosine hydroxylase (TH), tryptophan hydroxylase (TPH), all isoforms of nitric oxide synthases (NOS), and alkylglycerol monooxygenase (AGMO). These enzymes will be responsible for the production of the semi-essential amino acid tyrosine, the aminergic neurotransmitters dopamine and serotonin, nitric oxide, and neuroactive lipids, respectively. The de novo pathway will generate new molecules of BH4 by consuming energy in the form of guanosine triphosphate. The salvage pathway will also generate new BH4 molecules, but in a more economical way, and the recycling pathway will maintain the levels of BH4. The main activators of the de novo pathway are pro-inflammatory mediators, such as interferon gamma (IFN-δ); tumor necrosis factor alpha (TNF-α); interleukin 1 beta (IL-1β); IL-6 (interleukin-6); and lipopolysaccharides (LPSs). Abbreviations: GTPCH: guanosine triphosphate cyclohydrolase; PTPS: 6-Pyruvolyl tetrahydropterin synthase; SPR: sepiapterin reductase; AR: aldose reductases; CRs: carbonyl reductase; BH2: dihydrobiopterin; qBH2: dihydrobiopterin quinoid; DHFR: dihydrofolate reductase; PCD: Pterin-4-alpha-carbinolamine; DHPR: dihydropteridine reductase (DHPR). BH4 is an obligatory cofactor for the activity of the aromatic. Source: Eichwald et al. (2023), Ref. [12], with permission.

BH4 is an enzyme cofactor essential for the synthesis of monoamine neurotransmitters, for the metabolism of phenylalanine and lipid esters, and for the production of nitric oxide (NO) [11]. The intracellular concentration of BH4 is strictly maintained at low levels by three finely tuned biosynthetic pathways: the de novo, recycling, and salvage pathways (Figure 1) (for a review, see [12]). The de novo BH4 pathway reduces guanosine triphosphate (GTP) into BH4 by the successive action of three enzymes: GTP cyclohydrolase I (GTPCH), 6-pyruvoyl-tetrahydrobiopterin (PTPS), and sepiapterin reductase (SPR) [13]. In the absence of SPR, non-specific reductases (aldose reductase (AR) and carbonyl reductase (CR)) can also generate BH4 [14,15,16]. SPR, AR, and CR also participate in the salvage pathway, which utilizes a metabolic intermediate previously formed in the de novo pathway, 6-pyruvoyl-tetrahydrobiopterin, to generate a non-stable intermediate that non-enzymatically forms sepiapterin. Sepiapterin is then metabolized by SPR or CR into BH2, which is further transformed into BH4 by the action of dihydrofolate reductase [17]. Under metabolic/physiologic demand, these two metabolic vias produce new BH4 molecules, being the de novo pathway dependent on energy, and the salvage pathway a more economic route to synthesize BH4 [13]. Conversely, after BH4 is used as a mandatory enzyme cofactor and transformed into quinonoid dihydrobiopterin (qBH2), the enzyme dihydropteridine reductase regenerate it into BH4 via the recycling pathway [18,19].

Under physiological conditions, GTPCH is the rate-controlling enzyme in the BH4 pathway. GTPCH is encoded by *GCH1*, which is positively regulated by a variety of inflammatory and oxidant mediators, including lipopolysaccharide (LPS) [20,21], interferon gamma (IFN-γ) [21], interleukin 1 beta (IL-1β) [22,23], tumor necrosis factor alpha (TNF-α) [23], hydrogen peroxide [24], and others. During inflammation, *GCH1* is markedly upregulated; however, levels of other constitutive downstream enzymes that mediate the de novo pathway (PTPS and SPR) are only slightly increased, leading to a pseudo-metabolic blockage and a consequent accumulation of neopterin [25]. Indeed, neopterin has long been used as a sensitive biomarker for innate immune system activation for multiple acute and chronic conditions [25,26].

### 1.2. BH4 in ASD

A robust body of evidence gathered from various clinical trials suggests that dysfunction in the BH4 pathway may impair the activity of peptidergic, monoaminergic, and NO-dependent neurons in individuals with ASD [27,28,29,30,31]. Dopamine, oxytocin, and NO are all mediators that play significant roles in social bonding, sexual function, and pleasure, often interacting with each other, particularly within the hypothalamus, where they can influence behaviors like arousal, relaxation, and bonding. Dopamine is primarily associated with reward and motivation [32], and oxytocin with pair bonding and social connection [33]. NO acts as a signaling molecule to facilitate or to inhibit the release of other neurochemicals, including oxytocin [34,35].

### 1.3. BH4 as a Modulator of Dopamine and Oxytocin Signaling in ASD

The dopamine hypothesis of ASD posits that dysfunctions in the midbrain dopaminergic system, particularly in the mesocorticolimbic and nigrostriatal pathways, contribute to the core behavioral features of ASD, such as social deficits and stereotyped behaviors [36]. This hypothesis suggests that hyper- or hypo-dopaminergic signaling within brain target regions—highlighting the importance of an optimal level of dopaminergic signaling (involving dopamine transporter function, receptor availability, mutations, polymorphisms, and genetic variants)—could lead to or exacerbate ASD-related behavioral changes [36]. Furthermore, preclinical data have shown that dopaminergic neurons can be modulated by oxytocin, often affecting social and affiliative behaviors as well as reward pathways within the neural system [37].

The overlapping roles of these two neuroregulators in prosocial behavioral responses have been supported by anatomical and immunocytochemical studies, which reveal that their receptor binding sites and neuronal fibers are present in the same brain regions, often in close proximity to one another [38]. Additionally, it has been demonstrated that hypothalamic oxytocin cells express dopamine receptors, suggesting direct regulation by dopamine [39]. In this framework, BH4 availability emerges as a modulator of both dopamine and oxytocin activity. Beyond its role as a mandatory cofactor in dopamine production, microdialysis data indicate that exogenous BH4 can elicit the release of dopamine, serotonin, and glutamate in the rat striatum [40,41]. Moreover, the survival of dopaminergic neurons has been linked to the activity of the receptor Nurr1, which has been shown to enhance the transcription of tyrosine hydroxylase (the rate-limiting enzyme in dopamine biosynthesis) and *GCH1* (a gene encoding GTPCH, the first enzyme in the de novo BH4 pathway) in both in vitro and in vivo studies [42,43].

The unique properties of BH4 can be grouped with other physiological functions of BH4 in cell biology, as reported by our group over the past decade, including its capacity to enhance memory and cognition, support mitochondrial activity, and increase resistance to oxidative stress and inflammation [12,21,44,45,46,47]. Meanwhile, we have demonstrated that reduced BH4 levels induce mitochondrial oxidative stress and compromise energy production in cells with high energy demands [12,44]. Consistent with this, dopaminergic neurons, which are highly sensitive to oxidative stress and heavily reliant on mitochondrial function to meet their energy requirements, are particularly vulnerable to low BH4 levels. Thus, insufficient BH4 not only negatively affects the physiological biosynthesis of dopamine but also disrupts dopaminergic cell homeostasis. Indeed, mitochondrial dysfunction and oxidative stress are well-recognized pathological mechanisms implicated in ASD [48,49]. Conversely, inflammation, which is also involved in ASD pathophysiology, can elevate BH4 levels, potentially leading to excessive dopaminergic or oxytocin-mediated signaling, as previously reported [36]. This triad—neuroinflammation, oxidative stress, and mitochondrial dysfunction—commonly observed in ASD, initiates a cascade of events, including the dissociation of cell–cell junctions between endothelial cells and cytoskeletal reorganization, ultimately resulting in endothelial injury and a breach of the blood–brain barrier (BBB) [50,51,52]. Indeed, the altered expression of genes associated with BBB integrity has been observed in the brains of individuals with ASD, alongside increased neuroinflammation [53]. Additionally, markers of BBB permeability, such as autoantibodies against brain endothelial cells, S100B protein, platelet endothelial adhesion molecule-1 (PECAM-1), and vascular cell adhesion molecule-1 (VCAM-1), have been found to be altered in individuals with ASD [54]

### 1.4. BH4 and NO in ASD

Abnormalities in BH4-linked NO signaling cascades may disrupt NMDA receptor-mediated glutamatergic neurotransmission. NO is a gaseous neurotransmitter that regulates cerebral blood flow and plays critical roles in neuronal intracellular signaling, ranging from metabolic regulation to dendritic spine growth [55]. However, excessive NO levels can be harmful, as NO reacts with superoxide to form peroxynitrite, inducing oxidative stress [55].

NO production is catalyzed by nitric oxide synthases (NOS; NOS I–III). NOSI and NOSIII are constitutively expressed, and primarily localized in the brain, skeletal muscle, and endothelial cells, respectively [56]. In contrast, NOSII is highly sensitive to modulation by inflammatory processes [56]. Mediators of the Th1 immune response, such as LPS, cytokines, hydrogen peroxide, and other factors, upregulate the expression, content, and activity of NOSII. This enzyme is predominantly expressed in immune cells, including peripheral macrophages, microglia, and astrocytes, which are also major sources of neopterin [21,57].

NOSII relies on the increased biosynthesis of BH4 to produce large quantities of NO. During persistent inflammation, the initial upregulation of BH4 production will support NOSII activity. However, sustained NO synthesis or insufficient BH4 regeneration can lead to NOSII uncoupling, resulting in the production of superoxide instead of NO [58]. This uncoupling, caused by limited BH4 availability, contributes to inflammation and persistent oxidative stress, primarily through the formation of peroxynitrite. Peroxynitrite, in turn, depletes BH4 stores and triggers GTPCH proteolysis [59], creating a deleterious, vicious cycle in ASD.

### 1.5. BH4 and Comorbidities in ASD

ASD is primarily associated with psychiatric comorbidities. The prevalence and types of these comorbidities can vary across different age groups and populations. Anxiety disorders, including generalized anxiety disorder and social anxiety disorder, are common, with studies indicating a significant risk increase compared to the general population [60,61,62]. Mood disorders, such as depression and bipolar disorder, also have a higher incidence in individuals with ASD [60,61]. Attention-Deficit/Hyperactivity Disorder (ADHD) is another frequently observed comorbidity, with prevalence rates varying widely but often reported as high [63,64,65]. Obsessive–Compulsive Disorder (OCD) is also noted as a common comorbidity, with some studies suggesting a neurodevelopmental link between ASD and OCD [66].

Impaired BH4 metabolism has been extensively linked to the psychiatric features associated with ASD due to its role in the production of key neurochemicals, such as serotonin, dopamine, and NO. For instance, BH4 levels have been found to be reduced with increased neopterin and NO levels in various psychiatric disorders, including anxiety, depression, schizophrenia, schizoaffective disorder, and OCD, where BH4-dependent serotonin, dopamine and norepinephrine signaling have been shown to be disrupted [67,68,69,70,71,72]. Although the levels of BH4 in ADHD have not yet been reported, there is a consensus that individuals with ADHD might benefit from interventions with BH4 [73]. In addition, inflammation has also been shown to be involved in psychiatric disorders, and as previously stated, the persistent activation of the immune system can further compromise BH4 availability. Furthermore, genetic perturbations in BH4 metabolism have been linked to psychiatric manifestations in individuals. For example, patients with BH4 metabolism defects often exhibit psychiatric symptoms, including depression and anxiety, due to the resultant neurotransmitter imbalances [74]. Thus, impaired BH4 metabolism can also be responsible for ASD-associated comorbidities.

To investigate the role of this metabolic pathway in the pathophysiology of ASD, we conducted a systematic review to analyze the presence of markers associated with the BH4 pathway in the biological fluids of individuals with ASD. Additionally, we aimed to gather evidence supporting the off-label use of BH4 as a novel pharmacological approach for managing ASD. This is particularly relevant, as pharmacological treatments for ASD are primarily palliative, addressing comorbidities rather than targeting the underlying condition, for which currently there is no cure.

## 2. Method

The design, analysis, and reporting of this systematic review adhered to PRISMA 2020 guidelines [75]. The present systematic review was not registered online while it was in the planning stage. This of course increases the probability of an unplanned duplication.

### 2.1. Search Strategy

The databases utilized for research were PUBMED, SCIELO, COCHRANE, and BVS. We searched studies from January 1967 to December 2021 using the following search terms: BH4, Tetrahydrobiopterin, Neopterin, Nitric Oxide, and NO (only in COCHRANE and PUBMED) as metabolites of the BH4 pathway, combined with ASD, Autism, and Autism Spectrum Disorder. Non-original articles, such as other reviews and case-reports, were excluded. Articles in English, Spanish, and Portuguese were considered.

### 2.2. Eligibility Criteria

**Figure 2:**
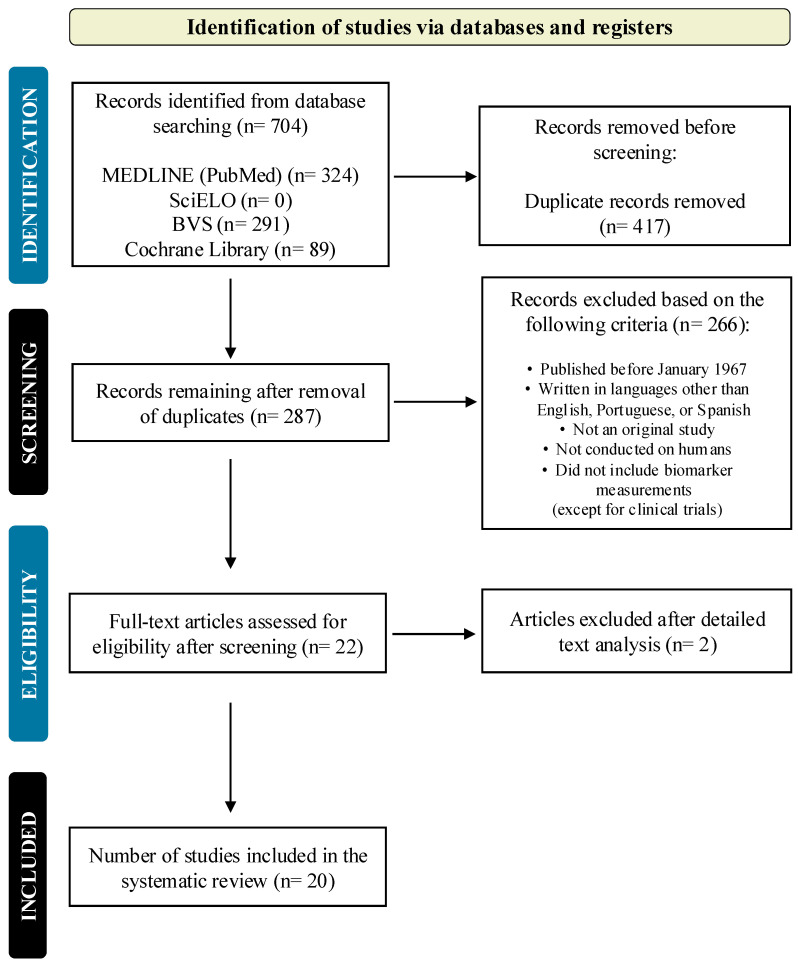
Flowchart for the selection of included studies. Nineteen studies were included in the review, as shown in the chart. The PubMed, BVS (Biblioteca Virtual em Saúde), Cochrane Library, and SciELO (Scientific Electronic Library Online) databases were used to identify the potential records.

To be included in the review, peer-reviewed studies must have been conducted on children and could include observational designs (e.g., cross-sectional, case–control studies) or experimental designs, such as randomized clinical trials. Eligible studies measured levels of BH4, neopterin, or nitric oxide in the biological fluids of individuals with ASD, with or without comparison to a control group. Data extracted from each study included the first author, publication year, study design, characteristics of the study population (e.g., target population, age, sex, sample size, comorbidities), control group details, and dosage or levels of BH4 (or reduced biopterin), nitric oxide (or nitrite/nitrate), neopterin (including descriptions of oxidized/reduced species), and other metabolites involved in the BH4 pathway in any fluid. Additionally, we also included BH4 treatment responses in case–control or randomized clinical trials that measured BH4 pathway metabolites. Studies that did not measure BH4, neopterin, or nitric oxide in the ASD population were excluded from the analysis, even if they involved BH4 treatment. The study selection process and progression are illustrated in Figure 2.

### 2.3. Selection Process

The literature search and selection process for this systematic review were conducted independently by three authors (LT, MM, and SR). This step determined whether the records met the inclusion criteria and filtered out studies that did not match the research needs. Any inconsistencies encountered during the process were solved through discussion with additional authors (GT, MAO, and LC). The complete selection process, from the initial number of studies screened to the final number included in the review, is reported and shown in the PRISMA flow diagram presented in Figure 2. This PRISMA flow diagram provides a summary of the information flow through the different stages of the systematic review, including screening, eligibility checks, and study inclusion.

### 2.4. Data Collection Process and Data Items

Data extraction was performed by four other authors (LM, LS, MO, and TN). The extracted data were cross-checked for accuracy and consistency by the first author (CC). This process ensured the reliability of the collected data and minimized the risk of errors. Any inconsistencies encountered during the process were referred to three additional authors (MAO, GT, and LC) for discussion and resolution in consultation with the corresponding author (AL). Collected data included age, sex, size of the sample, the inclusion of a control group, type of biological sample, levels of BH4 and related metabolites (neopterin and NO), methods employed for the quantitation of these metabolites, specifics of the study design (such as type of clinical study and intervention), behavioral parameters in ASD participants, and any correlation between clinical and BH4-related biochemical parameters. All included studies assessed BH4 levels using high-performance liquid chromatography (HPLC). This is the gold standard method for measuring BH4, which is normally coupled with electrochemical detection. However, other detection systems have been used, such as fluorescence detection and mass spectrometry. This approach is highly sensitive, specific, and widely used in clinical and research laboratories. The selected studies also used HPLC with fluorescent detection or ELISA for neopterin quantitation, and colorimetry or specific sensors based on chemiluminescence for measuring NO levels.

### 2.5. Risk of Bias

The risk of bias for each of the twenty included studies was assessed using RobVis, a web-based application designed specifically for visualizing risk-of-bias assessments within systematic reviews [76]. This tool was chosen for its ability to communicate the results of bias assessments through its visualization features. RobVis provides two main types of visual outputs. The first one, “Traffic Light” plot (Supplementary Figure S1), displays the domain-level judgments for each study, using a color-coded system where green indicates a low risk of bias, yellow indicates some concerns, and red indicates a high risk of bias. This visualization allows for an immediate grasp of the areas where biases may exist within and across studies. The second type of output is the weighted bar plot (Supplementary Figure S2) that is intended to show the distribution of risk-of-bias judgments within each bias domain across all included studies, weighted by the importance of each domain. This helps in understanding the overall landscape of biases that might affect this review’s findings.

### 2.6. Effect Measures

We used standardized mean difference (SMD) effect sizes (Cohen’s d) and their 95% confidence intervals (CIs) for continuous data when BH4 intervention was performed (before versus after intervention), or when a control group was compared to ASD participants.

### 2.7. Synthesis Methods

All 20 studies were included in the analysis. Means or standard deviations (SDs) were calculated when not presented in the studies. By using these data, we also calculated the CI and Cohen’s d. We used an octothorpe in Table 1, Table 2, Table 3 and Table 4 to indicate when data were calculated.

### 2.8. Treatment of Data Published in the Included Studies

To enable comparison across all the studies included in this review, we transformed the values presented in the records to standardize the data. For example, age was reported in month or years, and we standardize it to years in Table 1, Table 2, Table 3 and Table 4. BH4 levels were reported as ng/mL, pmol/mL, and nmol/L, and we standardized the presented values to nmol/L in Table 1, Table 2, Table 3 and Table 4.

### 2.9. Statistical Analysis and Certainty Assessment

As mentioned above, when absent, mean, SD, and CI were calculated, Cohen’s d was also calculated to estimate the magnitude of BH4’s effect when comparing two groups, according to published guidelines [77]. Cohen’s d helps to understand the magnitude of the difference between groups beyond just statistical significance. According to Cohen, d = 0.2 represents a small effect size, d = 0.5 a medium effect size, and d = 8 or higher a large effect size.

## 3. Results and Discussion

Our search through the referenced platforms yielded 704 records. After a thorough screening and removal of duplicates, we identified 19 records that met the inclusion criteria for this systematic review. The characteristics of these studies were organized based on their primary focus—whether it was on BH4, neopterin, or NO—as outlined in Table 1, Table 2, Table 3 and Table 4.

### 3.1. Characteristics of the Studies

The studies included in this systematic review used the Diagnostic and Statistical Manual of Mental Disorders, Fourth Edition, Text Revision (DSM-IV-TR) [1] as the primary diagnostic tool for ASD. Additionally, the diagnosis was further supported by administering the Childhood Autism Rating Scale (CARS) questionnaire [78], which, in some cases, was validated for different ethnic groups.

### 3.2. BH4 Levels in ASD

Table 1 summarizes the concentrations of BH4 in the biological fluids of children with ASD. Most studies reported lower BH4 levels in this population when compared to controls and to values reported in other studies [79,80]. Specifically, two studies (assessing BH4 in the cerebrospinal fluid; CSF; **Id. 2** and **5**) identified small-to-large effect sizes for reduced BH4 levels in ASD when compared to controls [81,82,83]. The reported values were below the lower limit of the normal range for CSF BH4 concentrations in children, which is considered to be around 30–50 nmol/L [79,80]. Conversely, two studies found greater CSF BH4 levels in children with ASD compared to controls or to previously published data [80,84].

**Table 1:** Levels of tetrahydrobiopterin in biological fluids of individuals affected by Autism Spectrum Disorder (ASD) [80,81,82,83,84,85].

Id.	Author (Year)	ASD n (Age; y)	Controls n (Age; y)	Biological Fluids	BH4 Levels in ASD (nmol/L)	CI 95%	BH4 Levels in Controls (nmol/L)	CI 95%	Effect Size (d)
1	Frye (2010)	21 (4.3)	-	CSF	Cluster 1 (n = 14): 20.14 ± 0.16	[20.05, 20.23] ^#^	-	-	-
					Cluster 2 (n = 7): 39.43 ± 1.24	[38.19, 40.67] ^#^	-	-	-
2	Tani et al. (1994)	20 (7.5)	10 (5.7)	CSF	5.60 ± 1.10	[5.11, 6.08] ^#^	13.50 ± 3.10	[11.28, 15.72] ^#^	3.99
3	Eto et al. (1992)	16 (12.3)	11 (10.4)	Plasma	1.24 ± 1.80 ^#^	[0.28, 2.20] ^#^	1.49 ± 0.52	[1.16, 1.82] ^#^	0.18
			12 (10.4)	Urine	0.74 ± 0.32 ^#^	[0.57, 0.91] ^#^	0.76 ± 0.17 ^#^	[0.67, 0.85] ^#^	0.07
4	Danfors et al. (2005)	12 (5.3)	-	CSF	26.08 ± 6.19 ^#^	[22.14, 30.02] ^#^			
5	Komori et al. (1995)	14 (4.2)	18 (4.9)	CSF	24.00 ± 10.45	[17.96, 30.04] ^#^	28.31 ± 11.90	[6.41, 40.21] ^#^	0.24
6	Fernell et al. (1997)	6 (4.1)	-	CSF	8.67 ± 2.75 ^#^	[3.29, 4.07]	-	-	

Abbreviations: Id: Identification of the record; ASD: Autism Spectrum Disorder; n = Sample size; Age: Age is shown as average; CSF: Cerebrospinal fluid; BH4: Tetrahydrobiopterin; CI: confidence interval; d = Effect size was calculated according to Cohen’s guidelines, where d = 0.2 indicates a small effect, d = 0.5 a medium effect, and d = 0.8 or larger, a large effect). ^#^: The values were calculated based on the information in the record to standardize the data. Levels of BH4 are indicated as mean ± standard deviation.

**Id. 1** found two clusters of ASD-affected children: cluster 1 presented lower values of BH4 in the CSF [80] and cluster 2 normal levels of pterin. The authors attributed these differences to the age of the participants. Participants in cluster 1 were older (5.50 ± 1.26 y) compared to those in cluster 2 (2.05 ± 0.36 y). While there is evidence of age-related changes in CSF BH4 levels in healthy children, direct evidence regarding age-related changes has been addressed in the literature by only one study [79]. The report showed that CSF BH4 levels vary by age, with peak levels in the first three months of life. After that, levels are lower until they plateau around age five [79]. For example, study **Id. 1** reported a negative correlation between age and CSF BH4 levels [80]. The second study that showed no differences in pterin levels (**Id. 3**) were found in the plasma and urine of the participants [85]. This study did not measure BH4 directly but instead quantified the oxidable species. It is known that during the oxidation process, all reduced forms of pterins are converted into biopterin [86], potentially masking any decrease in BH4 levels. Moreover, the reduced forms also involve a quinoid form of partially oxidized BH4. Interestingly, study **Id. 4** also identified two groups of participants in their cohort: one with BH4 levels above 30 nmol/L (considered within the normal range) and another with levels up to 30 nmol/L [84]. Participants in the latter, who had lower BH4 levels, were included in a double-blind, placebo-controlled crossover study. However, the levels of BH4 were not assessed after the intervention [84]. Id. 6 also reported very low levels of BH4 when compared with the reported values in the literature [79,80].

Although most of the studies presented in Table 1 did not include a control group to compare baseline BH4 levels, previous published data of normal BH4 levels [79,80] suggest that individuals with ASD tend to have lower levels of this pterin in the CSF.

Table 2 presents the effect of BH4 administration in the ASD population on behavioral parameters. A total of three published articles and three clinical trials with treatment data are included. These studies showed that children with ASD exhibited significant improvements across various behavioral scales, with all studies reporting enhancements in language and communication. Adverse effects were reported in all clinical trials, with irritability, sleep disturbances, and agitation being the most mentioned. The BH4 dose varied from 1 to 20 mg/kg/day across the studies.

**Table 2:** Effect of BH4 administration on behavioral parameters in individuals affected by Autism Spectrum Disorder (ASD) [27,28,29,81,83,84].

Id.	Author (Year)	Clinical Study	Clinical Trial Identifier	BH4 Group n (Age)	Placebo Group n (Age)	BH4 Dose (mg/kg/Day)	Baseline CSF BH4 Levels (nmol/L)	After Treatment CSF BH4 Levels (nmol/L)	Weeks of Intervention	Behavioral Parameters	Improvements	Adverse Effects (% BH4 Group)
4	Danfors et al. (2005)	Double-blind, randomized, placebo-controlled, cross-over design	-	6 (5.7)	6 (4.9)	3 (twice/day)			26 (alternating with placebo)	CARS	Improvement in social interaction in high-functioning young ASD children	Agitation and sleeping problems (83%)
5	Komori et al. (1995)	Case-Control study	-	14 (4.2)	18 (4.9)	1	C: 28.31 ± 11.90 ASD: 24.00 ± 10.45	ASD-R (n = 7): 20.56 ± 8.12 ASD-NR (n = 7): 27.40 ± 12.10	24	ABC	Slight improvement in language and communication, in eye contact and desire to interact, and in the number of words or sounds which the child used, mainly in individuals with lower BH4 levels. Improvement in social relationships’. DQ only improved in 1 participant, 70 to 83.	Pollakiuria (21%)
6	Fernell et al. (1997)	Pre-post intervention	-	6 (4.1)	-	3 (twice/day)	8.67 ± 2.75 ^#^	13.35 ± 2.90 ^#^	12	Griffiths Scale, PASS, DQ	Griffiths Scale, and PASS mainly in eye contact and desire to interact, and in the number of words or sounds which the child used, mainly in individuals with lower BH4 levels	Agressiveness and sleeping disturbances (16–50%)
7	Klaiman et al. (2013)	Double-blind, placebo-controlled trial	NCT00850070	23 (5.0)	23 (5.0)	20	-	-	16	CGI, ABC, SRS, PLS, VABS	Secondary measures indicated significant improvements for BH4 relative to placebo with regard to social awareness, autism mannerisms, hyperactivity, and inappropriate speech. effects were minimal and similar between both active medication and placebo.	Irritability (22%) Difficulty sleeping (9%) Repetitive behavior (4%) Hyperactivity (9%) Transient viral rash (9%)
8	Elliott et al. (2018)	Open-label extension study available only to subjects who completed an earlier double-blind, placebo-controlled study (Id. 7) of sapropterin in children with autism	NCT00943579	20 * (5.0)	21 * (5.0)	20	-	-	16	VABS, PLS, ABC	Improvements in ABC and SRS. Significant improvements in social awareness and social communication. A subset of children classified as “responders” exhibited notable improvements in behavior based on caregiver-reported assessments.	Irritability (20%) Bowel movement changes (25%) Repetitive behavior (25%) Difficulty sleeping (25%)
9	Frye et al. (2013)	Openlabel study	NCT01141595	10 (N/A)	-	20	-	-	16	PLS, VABS, SRS, ABC, ASQ, PCIS	Overall improvements in subscales of PLS, VABS ABC, ASQ	Irritability, excitement and mild upset stomach (10%), and Insomnia (10%)

* 15 completed the trial. Abbreviations: Id: Identification of the record; BH4: Tetrahydrobiopterin; Age: Age is shown as average; ASD: Autism spectrum disorder; C: Controls; ASD-R: ASD responders; ASD-NR: ASD non-responders; CGI: Clinical global impression scale; PASS: Pediatric autism severity scale; DQ: Developmental quotient; CARS: Childhood autism rating scale; ABC: Aberrant behavior checklist; SRS: Social responsiveness scale; PLS: Preschool language scale; VABS: Vineland adaptive behavior scale; ASQ: Autism symptoms questionnaire; PCIS: Parent-child interaction scale. ^#^: The values were calculated based on the information in the record to standardize the data. Levels of BH4 are indicated as mean ± standard deviation.

Study **Id. 5** reported that BH4 levels were lower in the CSF of ASD individuals before the pharmacological intervention (Table 1) [84]. After 24 weeks of BH4 treatment, the metabolite levels in both groups became similar, indicating that the intervention effectively restored BH4 levels. However, 50% of the participants were classified as responders based on CARS, which identifies the severity of autism [78]. This relatively low success rate has been reported in other studies using a BH4 dose of 1 mg/kg/day [87,88], indicating that higher doses may be necessary for individuals who are less responsive to the treatment.

Studies **Id. 4** and **6** used 3 mg/kg/day twice a day [83,84], and they reported that most of the individuals showed improvements, assessed by the Griffiths Developmental Scales (before starting and 3 months after completing the treatment) and the Parental Satisfaction Survey (PASS; assessed every fourth week). The Griffiths Developmental Scales assess the developmental progress from birth to 8 years old [89]. These scales help to identify delays or atypical development in areas such as cognition, motor skills, language, social and emotional development, and adaptive behavior. Additionally, few participants showed a clear increase in the developmental quotient (DQ), which measures a child’s developmental level relative to chronological age [90] (Table 2).

Three studies reported positive behavioral outcomes with a higher dose of BH4. Studies **Id. 7–9** used a dose of 20 mg/kg/day [27,29], which is the same dose used to treat phenylketonuria or genetic BH4 deficiencies, the conditions for which BH4 was originally approved by the Food and Drug Administration. These studies measured primary outcomes via the Clinical Global Impressions Improvement and Severity Scales (CGI-I) [91], which are widely used in clinical trials and research to assess the severity of illness, global improvement, and therapeutic response, as well as the Preschool Language Scale (PLS) [92]. For secondary outcomes, the Social Responsiveness Scale (SRS) and the Aberrant Behavior Checklist (ABC), assessed with the Vineland Adaptive Behavior Scales (Vineland) [93], autism symptoms questionnaire (ASQ) [94] and parent–child interaction scale (PCIS) [95], were used. **Id. 7–8** did not show changes in CGI-I [27,28,29]; however, they resulted in significant improvements for BH4 relative to placebo with regard to social awareness, autism mannerisms, hyperactivity, and inappropriate speech. Since these studies did not quantify BH4 levels in the plasma or CSF, it is difficult to correlate these effects to the availability of BH4 in the central nervous system. **Id. 9** used PLS as a primary outcome measure, which comprises receptive, expressive, and total communication skills [92]. Secondary outcome measures included assessments of subscales such as receptive, expressive, and written communication; personal, domestic, and community daily living skills; interpersonal relationships; play; and coping skills. The authors reported significant improvements in most of these scales following the intervention. **Id. 9** also found that BH4 supplementation did not change overall BH4 levels but increased the reduced-to-oxidized pterin ratio, enhancing its bioavailability. This increase is crucial for nitric oxide (NO) formation, as it is known that an improved ratio restores the coupling of NO synthases and supports the recycling of the trihydrobiopterin cation radical back to BH4 (for a review, see [12]).

Overall, BH4 supplementation shows promise, particularly at higher doses (20 mg/kg/day) and with extended treatment durations, in improving social, behavioral, and language outcomes in individuals with autism. However, the limited number of studies, small sample sizes, and lack of placebo-controlled or comparative trials highlight the need for larger, well-designed studies to better understand the impact of restoring BH4 levels in the central nervous system.

### 3.3. Neopterin Levels in ASD

Table 3 shows the concentrations of neopterin in three different fluids: urine, blood/plasma, and CSF. The studies reported a significant decrease in CSF neopterin concentrations in children with ASD. Two articles found a large effect size for elevated plasma neopterin levels in the ASD population, while in urine, one article reported a large effect size for increased neopterin levels, whereas another showed a decrease. Neopterin concentrations were measured using either ELISA kits or HPLC. Overall, the data suggest a tendency for central neopterin levels (neopterin in the CSF) to be diminished, while peripheral neopterin levels (in plasma and urine) tended to be elevated.

**Table 3:** Levels of neopterin in biological fluids of individuals affected by Autism Spectrum Disorder (ASD) [82,85,96,97,98,99].

Id.	Author (Year)	ASD n (Age)	Controls n (Age)	Method	Neopterin Levels in ASD (nmol/L)	Neopterin Levels in Controls (nmol/L)	Effect Size (d)
**Cerebrospinal fluid**							
2	Tani (1994)	20 (7.5)	10 (5.7)	HPLC	13.6 ± 1.50	15.6 ± 1.80	1.21
10	Zimmerman et al. (2005)	12 (5.8)	15 (7.5)	HPLC	8.18	16.80	-
**Plasma**							
3	Eto et al. (1992)	16 (12.3)	12 (10.4)	HPLC	2.89 ± 1.40 ^#^	6.04 ± 5.50 #	0.79
11	Zhao et al. (2015)	80 (3.7)	80 (3.7)	ELISA	7.87 ± 2.01	5.18 ± 1.31	1.58
Urine *							
3	Eto et al. (1992)	16 (12.3)	12 (10.4)	HPLC	390 ± 160 ^#^	620 ± 44.9 ^#^	1.96
12	Harrison et al. (1995)	17 (3–21)	17 (3.5–14.5)	HPLC	1306 ± 786	615 ± 373	1.12
13	Messahela et al. (1998)	14 (3–5)	16 (3–5)	HPLC	3116 ± 686	908 ± 201	4.37

Abbreviations: Id: Identification of the record; ASD: Autism Spectrum Disorder; n = Sample size; Age: Age is shown as average or range; HPLC: High-performance’ liquid chromatography; d = Effect size was calculated according to Cohen’s guidelines, where d = 0.2 indicates a small effect, d = 0.5 a medium effect, and d = 0.8 or larger, a large effect). Levels of neopterin are indicated as mean ± standard deviation, or standard error of mean (SEM) when indicated. * Urine neopterin levels are indicated in umol/mol creatinine. # Values extracted from the recod were transformed in nmol/mmol creatinine. ^#^: The values were calculated based on the information in the record to standardize the data. Levels of BH4 are indicated as mean ± standard deviation.

Studies **Id. 2** and **10** reported reduced neopterin levels in the CSF of children with ASD [82,96]. This aligns with the data presented in Table 1, which shows lower BH4 levels in ASD. Neopterin production is directly linked to the activity of the de novo BH4 pathway, which is responsible for the energy-dependent synthesis of new BH4 molecules (for a review, see [12]). Previous studies, including works from our group, have suggested that CSF neopterin levels reflect the central nervous system production of this metabolite [21,45,100]. These findings strongly indicate that BH4 metabolism is disrupted in the central nervous system of individuals with ASD. As a consequence, BH4-dependent metabolisms, i.e., the synthesis of the neurotransmitters dopamine, serotonin, and NO, and the processes they support will be compromised. BH4 is also mandatory for the production of the semi-essential amino acid tryptophan (precursor of serotonin and kynurenines) and for the synthesis of brain active lipids (for a review, see [12]). Deficiencies in these metabolites have been associated with several neurological alterations. Indeed, impaired tryptophan and kynurenine metabolisms have been associated with mood disorders such as depression, anxiety, and bipolar disorders [101,102], which are common comorbidities in ASD [61]. Moreover, mutations in AGMO that would compromise its catalytic activity have been identified to be present in individuals with ASD [103,104]. AGMO is an enzyme that metabolizes ether lipids in a BH4-dependent manner, and it is thought to produce bioactive lipids, which are essential for neurotransmission [104]. Furthermore, impaired dopamine transport and receptor availability (e.g., D1 and D2 receptors), or the presence of genetic variants in dopamine-related genes (such as DRD4, COMT, and DAT1) that compromise dopaminergic neurotransmission, have been associated with ASD traits [36].

Studies **Id. 3** [85] and **11–13** [97,98,99] presented results regarding neopterin levels in the plasma and urine of individuals with ASD. In agreement with studies **Id. 2** and **10**, **Id. 3**, reported reduced biomarker levels in both fluids, suggesting a diminished metabolic flow toward BH4 biosynthesis. However, studies **Id. 11–13** showed increased levels of neopterin in ASD. This discrepancy is not necessarily contradictory, as the increased levels might reflect sustained inflammation. Neopterin has been used for more than six decades as a very sensitive marker of immune system activation (for a review, see [12]). The presence of increased levels of Th1-related cytokines or reactive species will stimulate BH4 production; however, considering that monocytes and macrophages are cells with a lower content of PTPS that would lead also to increased neopterin formation [105]. Indeed, persistent inflammation is a hallmark of ASD [8]. Altogether, dysregulation in these pathways may lead to a cascade of effects on brain development, synaptic plasticity, and neuronal signaling, contributing to the core symptoms of ASD.

### 3.4. NO Levels in ASD

Table 4 shows the concentrations of NO in children with ASD. All studies reported increased NO concentrations compared to controls, independent of the type of biological system used in the investigations—plasma and whole blood (**Id. 14**, **15**, **16**, and **17**; [106,107,108,109]), red blood cells (**Id. 18**; [110]), urine (**Id. 19**; [111]), and saliva (**Id. 20**; [112])—further supporting the notion that NO metabolism is systemically altered in ASD, as previously proposed. However, no measurements of NO in the CSF were found in the literature in relation to ASD. The effect size of these differences ranged from moderate to very large effects, suggesting that NO might be involved in ASD physiopathology.

**Table 4:** Levels of nitric oxide (NO)-related metabolites in biological fluids of individuals affected by Autism Spectrum Disorder (ASD) [106,107,108,109,110,111,112].

Id.	Author (Year)	ASD n (Age)	Controls n (Age)	Method	Biological Luids	NO Levels in ASD	NO Levels in Controls	Effect Size (d)
14	Essa et al. (2012)	19	19	Commercial kit	Plasma	2433 ± 247 unit/mg protein	1139 ± 115 unit/mg protein	6.72
15	Tostes et al. (2012)	24	24	Griess reaction	Plasma	47.30 ± 5.10 μmol/L	35.70 ± 4.20 μmol/L	2.48
16	Lakshmi Priya & Geetha (2011)	LFA = 15 (4–12) MFA = 15 (4–12) HFA = 15 (4–12)	45 (4–12)	Griess reaction	Blood	LFA = 12.60 ± 1.80 MFA = 10.10 ± 1.50 HFA = 11.60 ± 1.70 unit/dL	8.90 ± 1.30 unit/dL	LFA vs. C: 2.58 MFA vs. C: 0.88 HFA vs. C: 1.90
17	Sweeten et al. (2004)	29 (6.1)	27 (6.5)	Griess reaction	Plasma	48.80 ± 12.10 μmol/L	40.90 ± 8.30 μmol/L	0.76
18	Sögüt, et al. (2003)	27 (4.7)	30 (5.1)	Gries reaction	Red blood cells	1.62 ± 0.49 μmol/g Hb	0.91 ± 0.22 μmol/g Hb	1.89
19	Fu et al. (2019)	44 (2–7)	Adults: 28 (18–25) Children: 30 (3–7)	Chemioluminescence using a NO analyzer	Urine	Nitrate: 2.87 ± 0.27 mmol/L	Adults: 2.80 ± 0.36 Children: 4.56 ± 0.59 mmol/L	ASD vs. Children: 3.95
						Nitrite: 0.87 ± 0.11 µmol/L	Adults: 0.58 ± 0.10 Children: 0.59 ± 0.07 µmol/L	2.92
						Nitrite/Nitrate ratio: 0.35 ± 0.04 · 10^−3^	Children: 0.16 ± 0.02 · 10^−3^	5.76
20	Yao et al. (2021)	126 (2–10)	129 (2–10)	Chemioluminescence using a NO analyzer	Saliva	Nitrite: 4.97 ± 3.77 μmol/L	Nitrite: 2.66 ± 2.07 μmol/L	0.76

Abbreviations: Id: Identification of the record; ASD: Autism Spectrum Disorder; n = Sample size; Age: Age is shown as average or range; NO: Nitric oxide; LFA: Low-functioning autism; MFA: Medium functioning autism; HFA: High functioning autism; d = Effect size was calculated according to Cohen’s guidelines, where d = 0.2 indicates a small effect, d = 0.5 a medium effect, and d = 0.8 or larger, a large effect). Levels of NO are indicated as mean ± standard deviation. Levels of BH4 are indicated as mean ± standard deviation.

Increased NO levels in biological fluids have been extensively associated with both acute and chronic inflammation [113]. Activated macrophages produce high levels of NO in order to enhance phagocytic activity during inflammation. While increased NO can effectively target undesired microbes, parasites, or tumor cells, it may also compromise host cell viability and homeostasis due to the high reactivity of NO towards protein-bound iron. NO can inhibit critical iron-containing enzymes, including complexes of the respiratory chain, as well as enzymes involved in the Krebs cycle and DNA synthesis, resulting in DNA oxidation and fragmentation [114]. In this context, the dysregulation of multiple pro-inflammatory or anti-inflammatory cytokines has been reported in the blood and CSF of individuals with ASD. For example, a recently published meta-analysis, which included 17 studies with a total sample of 743 participants, showed significantly increased concentrations of IL-1β, interleukin-6 (IL-6), interleukin-8 (IL-8), IFN-γ, eotaxin, and monocyte chemotactic protein-1 in individuals with ASD [115]. Many of these cytokines are able to stimulate the production of BH4 to sustain the generation of NO, and therefore, the immune response. However, this phenomenon of persistent immune system activation may ultimately result in a reduced availability of BH4. Additionally, preclinical studies have shown that NO and its derivatives can directly interact with proteins, lipids, and metabolites, including neuromodulators—especially serotonin and dopamine—and thereby change their regulatory action on synaptic transmission [116,117,118], contributing to the pathophysiology of neurological disorders, including ASD [61]. Moreover, it has also been found that NO negatively affects synaptogenesis, as well as the glutamatergic and GABAergic systems in the cortex and the striatum, favoring the development of ASD-like behavioral deficits [5].

Collectively, these studies underscore the multifaceted role of BH4-related NO activity in modulating neurotransmission, influencing both the chemical integrity of neurotransmitters (glutamate, GABA, serotonin, and dopamine) and the responsiveness of neural circuits.

Finally, most of the studies presented in this systematic review were conducted with small cohorts, making comparisons of BH4 levels, sex, and age challenging, and resulting in very low statistical power. The lack of research specifically addressing sex and age differences in the BH4 pathway in ASD represents a significant gap in the literature. Expanding studies that consider both sex and age, and including larger, more diverse groups could provide valuable insights into whether males and females with ASD face different metabolic challenges related to BH4. Such studies could also inform the development of more personalized treatment approaches based on sex-specific differences.

## 4. Conclusions

This review highlights significant alterations in the metabolism of BH4 in individuals with ASD. While most studies indicate lower central BH4 levels in the CSF of ASD children, the results are heterogeneous. These differences may be attributed to age-dependent factors or the presence of polymorphisms known to reduce BH4 production [119]. Additionally, the identification of increased NO levels points to a disruption in BH4-dependent pathways in ASD, likely compounded by chronic immune activation and inflammation. Elevated peripheral neopterin concentrations, contrasted with reduced central levels, strengthen the notion that neopterin concentrations in the CSF are primarily brain-derived and not significantly influenced by BBB dysfunction [21,100,120]. This suggests that neopterin’s presence in the central nervous system may be regulated by mechanisms within the brain, potentially involving efflux transport processes that would require a high blood-to-CSF ratio. This hypothesis is supported by recent preprint studies showing that BH4 (and neopterin, which is more polar than BH4) exhibited low permeability in a BBB model composed of rat brain endothelial cells. This transport was classified as passive diffusion through the narrow gaps of endothelial tight junctions [121]. Indeed, low brain BH4 permeability has been a limiting factor in the treatment of phenylketonuria, a challenge recently addressed by the results of the first clinical trial using sepiapterin, a more stable molecule that is transported more efficiently across cellular membranes than BH4 [122]. Therefore, during inflammation, it is plausible that neopterin efflux transport at the BBB could be influenced not only by the associated increase in permeability observed in ASD, but also by the need to achieve a plasma-to-CSF ratio of at least 40, as previously proposed [120]. However, the positive effects of BH4 supplementation on behavior in ASD suggest that BH4 metabolism may be centrally compromised. Larger, well-controlled studies are essential to assess the therapeutic potential of targeting this metabolic pathway in ASD.

## Data Availability

All data and code are stored on a repository of the Open Science Framework (Latini, A. ASDxBH4 Available online: https://osf.io/tw9kd).

## Supplementary Materials

The following supporting information can be downloaded at: https://www.mdpi.com/article/10.3390/brainsci15020151/s1, Figure S1: Risk-of-bias assessment for the twenty included studies; Figure S2: Graph bar results of the risk-of-bias assessment.
