# Supplementary material for: Tetrahydrobiopterin and Autism Spectrum Disorder: A Systematic Review of a Promising Therapeutic Pathway
Source: Brain Sci. 2025 Feb 3;15(2):151. doi: 10.3390/brainsci15020151 (PMC11853471; doi:10.3390/brainsci15020151)
Supplement: Supplementary file 1 [file brainsci-15-00151-s001.zip › brainsci-3429665-supplementary.pdf]

Supplementary Materials: *Risk of bias assessment*: The risk of bias for each of the twent included studies was assessed using RobVis, a web-based application designed specifically for visualizing risk-of-bias assessments within systematic reviews. This tool was chosen for its ability to communicate the results of bias assessments through its visualization features. RobVis provides two main types of visual outputs. The first one, "Traffic Light" plot (**Supplementary Figure S1**), displays the domain-level judgments for each study, using a color-coded system where green indicates a low risk of bias, yellow indicates some concerns, and red indicates a high risk of bias. This visualization allows for an immediate grasp of the areas where biases may exist within and across studies. The second type of output is the weighted bar plot (**Supplementary Figure S2**) that Is intended to show the distribution of risk-of-bias judgments within each bias domain across all included studies, weighted by the importance of each domain. This helps in understanding the overall landscape of biases that might affect this review's findings.

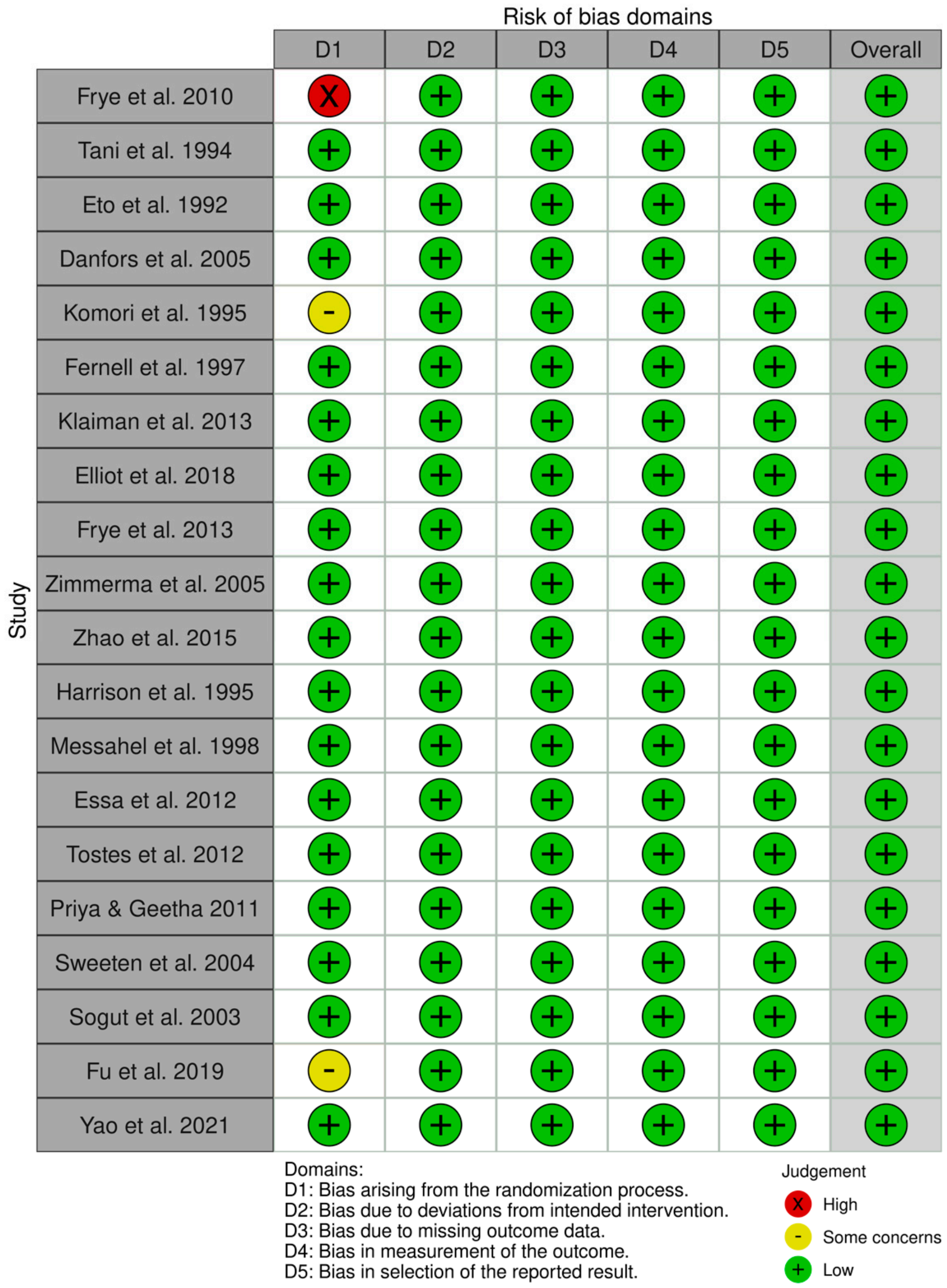

Supplementary Figure S1. Risk of bias assessment for the twenty included studies.

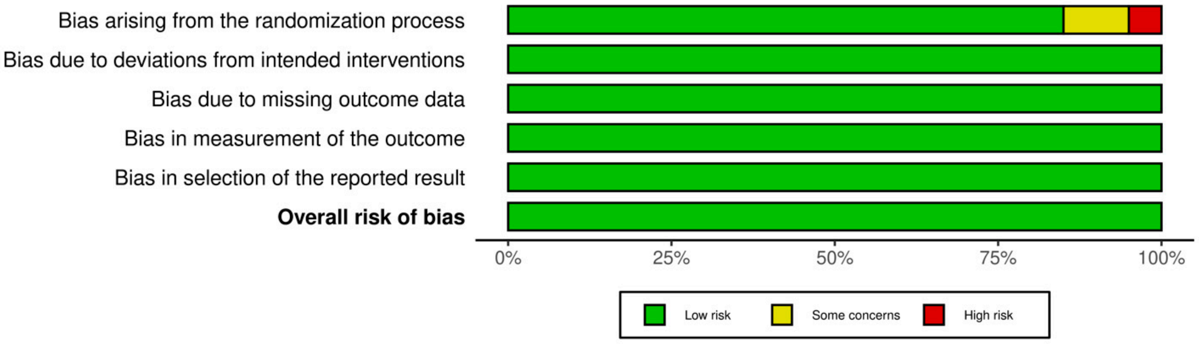

Supplementary Figure S2. Graph bar results of the risk of bias assessment.
